# Repurposing minocycline as an added-on treatment for ulcerative colitis patients on mesalamine: a randomized clinical pilot study

**DOI:** 10.1007/s00210-025-04662-2

**Published:** 2025-10-23

**Authors:** Amira F. Mashaly, Sahar K. Hegazy, Monir M. Bahgat

**Affiliations:** 1 Pharmacy Practice Department, Faculty of Pharmacy, Horus University-Egypt, New Damietta, 34518 Egypt; 2 Clinical Pharmacy Department, Faculty of Pharmacy, Tanta University, El-Guiesh Street, El-Gharbia Government, Tanta, 31527 Egypt; 3 Internal Medicine Department, Faculty of Medicine, Mansoura University, Mansoura, Egypt

**Keywords:** Ulcerative colitis, Minocycline, Mesalamine, ICAM-1, MMP-12

## Abstract

One of the most prevalent forms of chronic inflammatory bowel disease is ulcerative colitis (UC). The key characteristics observed in UC patients involve fecal urgency, abdominal discomfort, and bloody diarrhea, all of which significantly lower their quality of life. Preclinical studies investigated the protective effect of minocycline in animal models of colitis. This study aimed to assess minocycline’s potential efficacy and safety in mesalamine-treated UC patients. This randomized, controlled pilot clinical research included 46 individuals with mild to moderate UC who met the inclusion criteria. The mesalamine group (*n* = 23) received 1 g of mesalamine three times a day for 6 months. The minocycline group (*n* = 23) received minocycline 100 mg twice daily and mesalamine 1 g three times a day. Patients were evaluated by a gastroenterologist using the Short Inflammatory Bowel Disease Questionnaire (SIBDQ), Truelove and Witts Severity Index, Brief Pain Inventory (BPI), and the non-invasive Partial Mayo Score (PMS). Before and after 6 months of treatment, each patient’s levels of nitric oxide (NO), matrix metalloproteinase-12 (MMP-12), and intracellular adhesion molecule 1 (ICAM-1) were measured. Serum levels of MMP-12, ICAM-1, and NO were statistically lower in the minocycline group than in the mesalamine group. In the minocycline group, the Truelove and Witts Severity Index, PMS, and BPI pain intensity all significantly dropped, whereas SIBDQ was substantially elevated compared to the mesalamine group. Minocycline could serve as a potential adjunctive remedy for enhancing clinical outcomes, improving quality of life, and modulating inflammation in patients with mild to moderate UC.

**Trial registration**: ClinicalTrials.gov ID: NCT06201793. Trial registration date 22–1-2024.

## Introduction

Samuel Wilks was the first to identify ulcerative colitis (UC) in the 1800 s, along with Crohn’s disease. Constant inflammation of the colon’s mucosal layer, initiating at the rectum and progressing upward, is a hallmark of idiopathic inflammatory bowel disease (IBD). It is a chronic disease that typically emerges in early adulthood and is marked by symptoms such as bloody diarrhea and abdominal cramps (Feuerstein and Cheifetz 2014). Although the precise etiology of UC is still unknown, previous studies have suggested that genetic factors, diet, and environmental influences all play significant roles in its development (Ganji-Arjenaki and Rafieian-Kopaei 2019; Le Berre et al. 2023). A key factor that plays a role in both the onset and progression of UC is thought to be the disruption of the immunological responses of the intestinal mucosa (Neurath 2019). The intestinal mucosa serves vital functions, including mechanical, chemical, biological, and immune barriers. As a result, safeguarding the intestinal epithelium’s integrity and preserving the mucosal barrier’s healthy functions have become key concerns in present UC studies and treatment strategies (Li et al. 2021).

Oxidative stress plays a central role in the pathogenesis of ulcerative colitis (UC) and other inflammatory conditions (Andrabi et al. 2023; Muro et al. 2024). This state is characterized by an imbalance where the production of reactive oxygen species (ROS), like superoxide anion, hydrogen peroxide, and hydroxyl radicals, overwhelms the antioxidant system (Andrabi et al. 2023).

The body produces its own NO from l-arginine using a specific group of enzymes called NO synthases (NOS). NO acts as a key signaling molecule in several physiological functions, including vasodilation, regulating inflammation, and apoptosis (Andrabi et al. 2023). These various physiological roles of NO are facilitated by remarkably low concentrations (Miller and Megson 2007). On the contrary, higher NO concentrations promote oxidative stress as its cellular properties and targets are different, leading to cytotoxicity (Andrabi et al. 2023). In UC, iNOS is upregulated by pro-inflammatory signals, including tumor necrosis factor-α (TNF-α), interleukin (IL-1β), and bacterial products (Sabo et al. 2023), resulting in excessive NO that reacts with superoxide to form peroxynitrite, a potent oxidant. This further induces inflammation and mucosal destruction (Bahaa et al. 2024).

In IBD, intercellular adhesion molecule-1 (ICAM-1) facilitates leukocyte adhesion and migration into inflamed tissues, and its expression correlates with disease severity (Nielsen et al. 1996). El Mahdy et al. demonstrated that mice lacking ICAM-1 were protected from severe forms of experimentally induced colitis, highlighting the vital function of ICAM-1 in UC development (El-Mahdy et al. 2021).

Matrix metalloproteinases (MMPs) are enzymes that rely on zinc ions (Zn^2+^) to function in the regulation and degradation of the extracellular matrix (ECM), playing key roles in tissue remodeling, wound healing, and inflammation. Among them, MMP-12 (human macrophage elastase) has been linked to IBD, characterized by the disruption of epithelial tight junctions and an excessive inflammatory response (Nighot et al. 2021). MMP-12 breaks down ECM components such as elastin, type collagen, laminin, and fibronectin, and it is crucial for macrophage migration and contributing to mucosal damage (Nighot et al. 2021). Its involvement in inflammation and tissue remodeling makes it a potential target for developing therapies for treating UC and various inflammatory conditions (Nénan et al. 2005).

Minocycline (7-dimethylamino-6-desoxytetracycline) is a second-generation tetracycline antibiotic known for its beneficial pharmacological properties. While it has long been used as an antibiotic and treatment for acne, minocycline, as well as other traditional and modified tetracyclines, has recently gained significant scientific attention due to its emerging pharmacological effects. Beyond its traditional uses (Pourgholami et al. 2013), the drug repurposing approach was investigated in many diseases. Minocycline has been proposed in several studies, such as depression (Soczynska et al. 2012), Alzheimer’s disease (Chu and Praticò 2011), epilepsy (Nowak et al. 2012), stroke (Zhao et al. 2011), and even acquired immune deficiency syndrome (Zink et al. 2005). It is hypothesized that these therapeutic effects originate from a combination of properties, including the prevention of apoptosis, the reduction of oxidative damage, the elimination of free radicals, and the suppression of inflammation (Panizzutti et al. 2023). The anti-inflammatory effects of minocycline have been demonstrated through both in vivo and in vitro investigations. Specifically, Mesa et al. have provided evidence that it has an anti-inflammatory activity in rodent colitis models, possibly due to its antibacterial and immunomodulatory actions (Garrido-Mesa et al. 2011). Additionally, Yu Huang et al. revealed that minocycline protects mice against dextran sulfate sodium (DSS)– or trinitrobenzene sulfonic acid (TNBS)–induced colitis, likely by inhibiting the expression of iNOS and MMPs in intestinal tissue–induced colitis (Huang et al. 2009). Until now, there have not been any clinical studies evaluating the role of minocycline in patients with UC.

Building on these findings, the current study aims to investigate minocycline’s potential as a novel anti-inflammatory treatment for UC, specifically focusing on its effects on NO, ICAM-1, and MMP-12.

## Patients and methods

Between November 2023 and January 2025, 46 individuals who met the eligibility criteria were enrolled in this study. The research protocol was conducted after obtaining ethical approval from the National Research Ethics Committee of the Faculty of Medicine at Mansoura University (approval code: MS.23.10. 2569.R2). The study’s design and methods followed the ethical guidelines outlined in the Helsinki Declaration and its later revisions from 1964. Before participation, all individuals provided informed consent and were assured of their right to withdraw from the study whenever they wished. This was a randomized, controlled pilot study clinical trial.

### Inclusion criteria

Individuals aged 18 and above, regardless of sex, were eligible to participate in the study if they had a confirmed diagnosis of mild to moderate UC based on endoscopic evaluation and were receiving treatment with 5-aminosalicylic acid (mesalamine).

### Exclusion criteria

Patients were excluded from the study if they had severe ulcerative colitis, were pregnant or breastfeeding, had significant liver or kidney problems, had been diagnosed with colorectal cancer, or were using steroids either rectally or systemically. Additionally, individuals on immunosuppressants or biological therapies and patients taking antacids, anticoagulants, bactericidal antibiotics, oral contraceptives, and isotretinoin, as well as those with a history of alcohol or drug addiction, known allergies to the medications being studied, or a history of complete or partial removal of the colon, were also not included.

### Study design

**Fig. 1:**
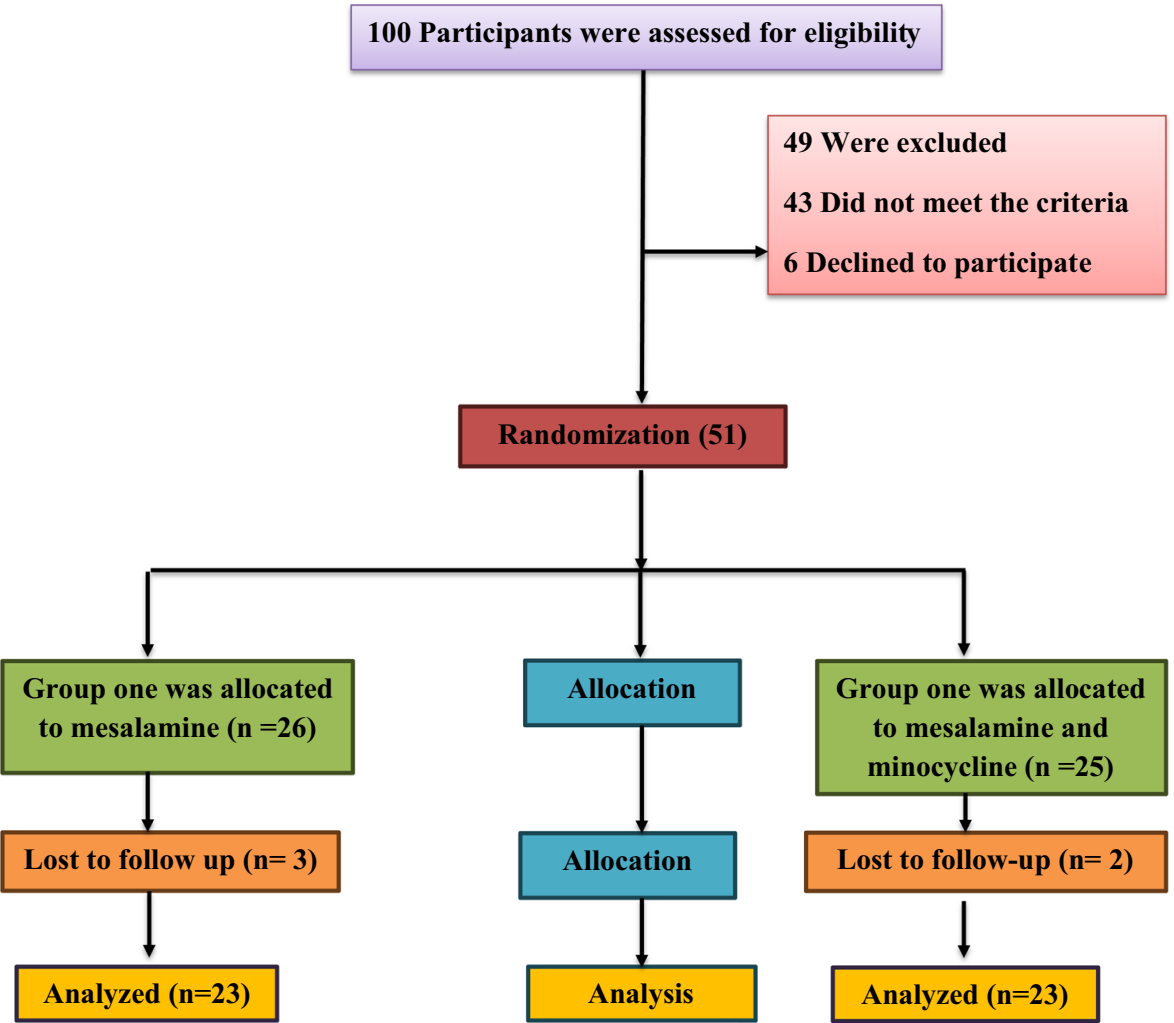
CONSORT diagram showing the flow of patients throughout the study

This clinical study, registered in 2023 (www.ClinicalTrials.gov with the number NCT06201793), was a randomized controlled study designed to assess the safety and efficacy of minocycline and mesalamine in managing UC. In Fig. 1, the CONSORT flow diagram shows that 23 participants who met the criteria and gave their written consent were randomly put into one of two groups (mesalamine and minocycline groups). This random assignment was done using a computer program to generate random numbers and blocks.

Both groups received 1 g of mesalamine tablets (Pentasa® 500 mg, Multi Pharm, Egypt) three times per day (t.i.d.) for 6 months. However, the minocycline group also received 100 mg of tablets twice daily (b.i.d.) (Minocin® 100 mg, Pfizer) during the same period.

### Sample size calculations

Since there are no previous clinical studies aimed at evaluating the effect of minocycline on ulcerative colitis or evaluating its anti-inflammatory activity, we used on G*Power software version 3.1.9.7 (2022 Heinrich-Heine-Universität Düsseldorf, Germany) to calculate the required sample size. It is estimated that a total sample size of 40 patients (20 patients in each group) could provide statistical power of 0.82 with the assumption that the attrition rate is 10% so the starting sample size is 46 patients (23 patients in each group).

### Study protocol

UC patients, after confirming their eligibility, received detailed neurological, physical, and psychological assessments. They were then randomly allocated to either a mesalamine-only group (1 g t.i.d.) or a combination group receiving mesalamine (1 g t.i.d.) plus minocycline (100 mg bid). All treatments were oral, and participants in both groups were provided with nutritional and lifestyle counseling. The chosen dosages for mesalamine (Sehgal et al. 2018) and minocycline were selected based on their established use and safety profile in previous clinical studies involving inflammatory and autoimmune conditions (Zhang et al. 2008; Metz, et al., 2017; Li et al. 2025). The 6-month treatment duration was based on both clinical relevance and practical considerations. UC is a chronic, relapsing condition, and sustained treatment is often required to observe meaningful clinical and biochemical improvements. Six months was considered sufficient to evaluate short- and intermediate-term outcomes, including symptom control, biomarker changes, and safety. This duration also aligns with standard follow-up intervals used in routine clinical practice and previous maintenance therapy trials (Metz et al. 2017a; Bahaa et al. 2024).

### Follow-up

Monthly in-person visits and phone conversations were performed to follow up with patients. During the initial visit, a comprehensive evaluation was performed, including kidney and liver function tests, complete blood counts, and a full medical history to exclude any underlying organ dysfunction. Additionally, serum levels of NO, ICAM-1, and MMP-12 were assessed at baseline and following 6 months of treatment intervention.

### Evaluation of colitis

The severity of UC was evaluated using the Partial Mayo Score (PMS) index, a non-invasive clinical tool. This index assesses UC activity through three components: stool frequency, rectal bleeding, and the physician’s global evaluation (Lewis et al. 2008), with total scores ranging from 0 to 9. PMS scores were recorded at both the start and end of treatment. The criteria for a clinical response included achieving a rectal bleeding subscore of 0 or 1, a reduction of ≥ 1 point in that subscore, or a decrease of ≥ 2 points and ≥ 30% from the initial PMS. Clinical remission, on the other hand, was considered when the PMS was below 2, with no individual subscore exceeding 1 (Probert et al. 2018).

Furthermore, the Truelove and Witts Severity Index, a clinical tool employed to categorize the severity of UC into mild, moderate, and severe based on six variables: daily stool frequency, blood in the stools, pulse rate, temperature, erythrocyte sedimentation rate (ESR), and hemoglobin levels (Hgb) (Truelove and Witts 1955). Mild disease was characterized by ≤ 4 daily bowel movements with minor visible blood, no fever or tachycardia, mild anemia, and ESR ≤ 30 mm/h (Pabla and Schwartz 2020). Severe disease included ≥ 6 daily bowel movements with macroscopic blood, fever (> 37.5 °C evening or ≥ 37.8 °C on ≥ 2 of last 4 days), heart rate > 90 bpm, severe anemia (hemoglobin ≤ 75%), and a significantly elevated ESR > 30 mm/h. Moderate disease represented a state intermediate between severe and mild disease (Pabla and Schwartz 2020; Truelove and Witts 1955).

### Pain Severity and Quality of Life Assessment

Pain is a subjective experience, and as such, its measurement relies on patients’ self-reports. Various tools and questionnaires have been created to evaluate pain, including numeric and verbal rating scales (Klepstad et al. 2002). One such tool is the Brief Pain Inventory (BPI) created by Cleeland and Ryan (1994). The BPI assesses pain through two main sections: one measuring pain severity using four questions and another evaluating pain’s interference with daily function through seven questions. For pain severity, patients rate their current, worst, least, and average pain over the last 24 h on a 0-to-10 scale (no pain to worst pain). The sum of these ratings creates a pain severity index. The seven interference questions ask patients to rate how much their pain affects general activity, walking, mood, sleep, work, relationships, and enjoyment of life, using a 0-to-10 scale (no interference to complete interference) (Klepstad et al. 2002). The sum of these scores yields a function interference index (Jelsness-Jørgensen et al. 2016).

The Short Inflammatory Bowel Disease Questionnaire (SIBDQ), a brief and self-completed tool developed by Irvine and colleagues, is reliable and effective in evaluating the health-related quality of life (HRQOL) in Crohn’s disease (CD) and UC patients, has gained widespread recognition, and is now commonly used in both clinical settings and research worldwide. It is ten questions that cover social, bowel, emotional, and systemic aspects (Irvine et al. 1996). Total scores on the SIBDQ range from 10 to 70, reflecting poor to optimal HRQOL, respectively. This score is derived from rating each of the questionnaire’s items on a 7-point Likert scale, where 1 signifies a severe problem and 7 signifies no problem. A total SIBDQ score below 50 is interpreted as indicative of a diminished quality of life (Roseira et al. 2021).

### Study outcomes

#### Primary outcomes

Changes in PMS were used as the primary endpoint to evaluate clinical remission and response in patients with mild to moderate ulcerative colitis.

#### Secondary outcomes

Secondary outcomes include changes in BPI and SIBDQ score, as well as measuring serum levels of different mediators such as NO, ICAM-1, and MMP-12.

#### Sample collection

Prior to the intervention and six months later, 10 mL of blood was drawn from a vein in the forearm (antecubital vein). The samples were transferred to tubes, left to coagulate, and then processed by centrifugation at 4500 g for 10 min using a Hettich Zentrifugen EBA 20 (Westfalia, Germany). Upon collection, the serum was divided into two aliquots. One was used for immediate assessment of hepatic and kidney function, while the other was stored at − 80 °C for cytokine level analysis at a later time.

#### Biochemical analysis

Liver enzyme levels of alanine aminotransferase (ALT) and aspartate transaminase (AST) in serum samples were assessed spectrophotometrically (Huang et al. 2006). Serum creatinine, an indicator of kidney function, was measured via the Jaffé reaction (Husdan and Rapoport 1968). Complete blood counts were determined from 2 mL EDTA blood samples using an automated hematology analyzer. Serum levels of nitric oxide (NO), ICAM-1, and MMP-12 were quantified using commercially available enzyme-linked immunosorbent assay (ELISA) kits (Sunred Biotechnology, Shanghai, China) according to the manufacturer’s protocols (catalogue nos.. 201–12–1511, 201–12–0264, and 201–12–5429, respectively).

#### Statistical analysis

Prism version 8 (GraphPad Software, Inc., San Diego, California, USA) was used for statistical analyses. The Shapiro–Wilk test checked for normality. Normally distributed data were compared between groups (unpaired *t*-test) and within groups (paired *t*-test) before and after treatment. Nonparametric data were compared using Mann–Whitney (between groups) and Wilcoxon (within groups) tests. Quantitative results are reported as mean ± SD and qualitative as counts, medians, and interquartile ranges. Correlations were assessed with Spearman or Pearson tests. Chi-square or Fisher’s exact tests were used to analyze categorical data between the treatment groups, while McNemar’s test was employed for within-group comparisons.

## Results

### Baseline demographic characteristics

The minocycline and mesalamine groups were comparable at baseline for AST (*p* = 0.2137), ALT (*p* = 0.1066), sex (*p* = 0.7672), age (*p* = 0.0789), BMI (*p* = 0.9523), SrCr (*p* = 0.5994), UC disease duration, and the disease location, which included proctitis (limited to the rectum), left-sided colitis (extending up to the splenic flexure), and proctosigmoiditis (involving both the rectum and sigmoid colon) as no statistically significant differences were found (Table 1).

**Table 1:** Clinical, demographic, and laboratory data of the patients

Parameter	Mesalamine group (*n* = 23)	Minocycline group (*n* = 23)	*p* value
Age (years)	45.96 ± 9.702	50.65 ± 7.912	0.0789
Sex (M/F)	12/11	13/10	0.7672
BMI (kg/M^2^)	23.11 (22.28–25.50)	23.34 (22.09–25.91)	0.9523
Serum ALT (IU/L)	36.65 ± 5.967	34.09 ± 4.492	0.1066
Serum AST (IU/L)	31.55 ± 6.815	33.91 ± 5.537	0.2137
SrCr (mg/dL)	0.9261 ± 0.1685	0.8957 ± 0.2184	0.5994
Disease duration	1.626 ± 0.5213	1.743 ± 0.5928	0.4827
Mild UC	14(60.87%)	13(56.52%)	0.7646
Moderate UC	9(39.13%)	10(43.48%)	0.7646
Site of disease (no)			
Proctitis	8	7	.999
Left-sided	7	6	.999
Proctosigmoiditis	8	10	0.7631

Data was presented as mean ± SD, median, and interquartile range and numbers; mesalamine group: ulcerative colitis patients treated with mesalamine1 g three times daily for 6 months; minocycline group: ulcerative colitis patients treated with mesalamine plus minocycline100 mg two times daily for 6 months

*M* male, *F* female, *BMI* body mass index, *AST* aspartate aminotransferase, *SrCr* serum creatinine, *ALT* alanine aminotransferase

Significance at *p* < 0.05

### Effect of study medications on clinical data

The data presented in Table 2 offer an in-depth examination of clinical markers, such as symptoms and disease activity scores, along with the effects of study medications on patient outcomes in individuals with mild to moderate UC. It compares the PMS, SIBDQ, and BPI scores before and after treatment in both the mesalamine and minocycline groups.

**Table 2:** Effect of study medications on clinical data

	Mesalamine group			Minocycline group			*p* value
Character	Before treatment	After treatment	*p* value	Before treatment	After treatment	*p* value	After treatment
**PMS** **response (*n*, %)** **Remission (*n*, %)**	4 (4–5)	2 (1–3)	< 0.0001^a^	4 (3–5)	1 (0–2)	< 0.0001^a^	0.033^b^
	13 (56.52%)			18 (78.26%)			0.042^c^
	10 (43.48%)			17 (73.91%)			0.036^c^
**SIBDQ**	39.74 ± 2.684	51.91 ± 2.392	< 0.0001^a^	41.17 ± 2.103	65.04 ± 3.240	< 0.0001^a^	< 0.0001^b^
**BPI (pain intensity)**	7.5 (6.8–7.6)	4.6 (4.1–5.2)	< 0.0001^a^	6.3 (6.1–7.3)	4 (3.1–4.2)	< 0.0001^a^	< 0.0001^b^
**BPI (pain interference)**	6.983 ± 0.189	3.374 ±.1888	< 0.0001^a^	5.926 ± 0.297	3.365 ± 0.149	< 0.0001^a^	0.863^b^

Data was presented as mean ± SD, number, median, and interquartile range. Mesalamine group: ulcerative colitis patients treated with mesalamine. Minocycline group: ulcerative colitis patients treated with mesalamine plus minocycline

*PMS* Partial Mayo score index, *BPI* Brief Pain Inventory, *SIBDQ* Short Inflammatory Bowel Disease Questionnaire

^a^Level of significance within the same group by paired *t*-test and Wilcoxon test for parametric and nonparametric data, respectively

^b^Level of significance between groups using unpaired* t*-test and Man Whitney test for parametric and nonparametric data, respectively

^c^level of significance between groups using Chi-square test. Significance at (*p* < 0.05)

The baseline values of the mesalamine and minocycline groups revealed no statistically significant differences (*p* > 0.05) when the unpaired *t*-test was employed for parametric variables and the Mann–Whitney test for non-parametric variables.

**Fig. 2:**
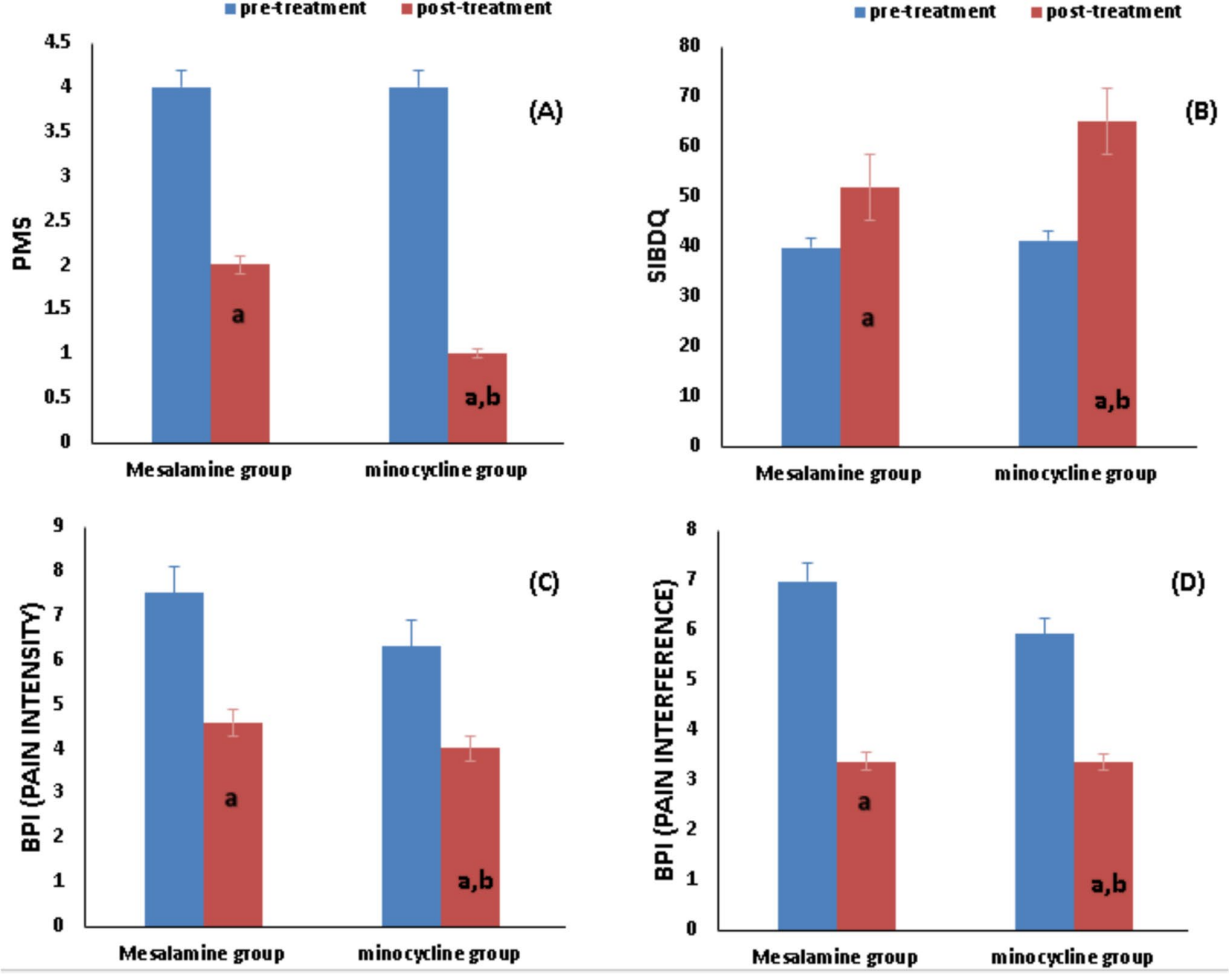
Effect of study medications on clinical data. Mesalamine group, ulcerative colitis patients treated with mesalamine; minocycline group, ulcerative colitis patients treated with mesalamine plus Minocycline. Effect of drug treatments on (**A**) changes in PMS, (**B**) changes in SIBDQ, (**C**) change in BPI (pain intensity), and (**D**) change in BPI (pain interference). PMS, partial Mayo score, SIBDQ, Short Inflammatory Bowel Disease Questionnaire, BPI, Brief Pain Inventory. (a) Significance compared to before treatment. (b) Significance compared to group on the mesalamine group

The Wilcoxon test for the mesalamine and minocycline groups showed a substantial reduction in PMS [4 (4–5) versus 2 (1–3) (*p* < 0.0001) and [4 (3–5) versus 1 (0–2), *p* < 0.0001] respectively in comparison to the baseline Table 2 and Fig. 2.

After 6 months of management, PMS showed a statistically significant decrease (*p* = 0.033) according to the Mann–Whitney test (Table 2 and Fig. 2).

The mesalamine group’s PMS response rate was 56.52% (*n* = 13/23), and remission rate was 43.48% (*n* = 10/23), according to the chi-square test. The PMS response rate in the minocycline group was 78.26% (*n* = 18/23), and the remission rate was 73.91% (*n* = 17/23).

Both groups experienced statistically significant increases in the SIBDQ when compared to the baseline. According to Table 2, the mesalamine group’s paired *t*-test result was 39.74 ± 2.684 compared to 51.91 ± 2.392, *p* < 0.0001, while the minocycline group was 41.17 ± 2.103 versus 65.04 ± 3.240, *p* < 0.0001. Following 6 months of intervention, the unpaired *t*-test revealed statistically significant variations in SIBDQ between the two groups as follows: SIBDQ (*p* < 0.0001) (Table 2 and Fig. 2).

### Effect of study medications on Brief Pain Inventory

The Wilcoxon test displayed that there was a statistical reduction in median pain intensity in the mesalamine group in comparison with baseline [7.5(6.8–7.6) versus 4.6(4.1–5.2) (*p* < 0.0001)]. While the reduction in median pain intensity in the minocycline group was [6.3(6.1–7.3) versus 4(3.1–4.2) (*p* < 0.0001)]. There was a statistically significant difference in pain intensity between the two groups (*p* < 0.0001), as shown by the Mann–Whitney test (Table 2).

Compared to baseline, the mesalamine and minocycline group’s paired *t*-test showed that there was a statistical drop in pain interference (6.983 ± 0.189 versus 3.374 ± 0.1888 (*p* < 0.0001)), and (5.926 ± 0.297 versus 3.365 ± 0.149, *p* < 0.0001) respectively (Table 2). Pain interference did not change significantly between the two groups after 24 weeks of treatment, as determined by an unpaired *t*-test (*p* = 0.863) (Table 2 and Fig. 2).

### Effect of study medications on disease severity according to the Modified Truelove and Witt’s Classification

The unpaired *t*-test and Mann–Whitney for parametric and non-parametric data revealed no significant difference (*p* > 0.05) in baseline values between the minocycline and mesalamine groups.

**Fig. 3:**
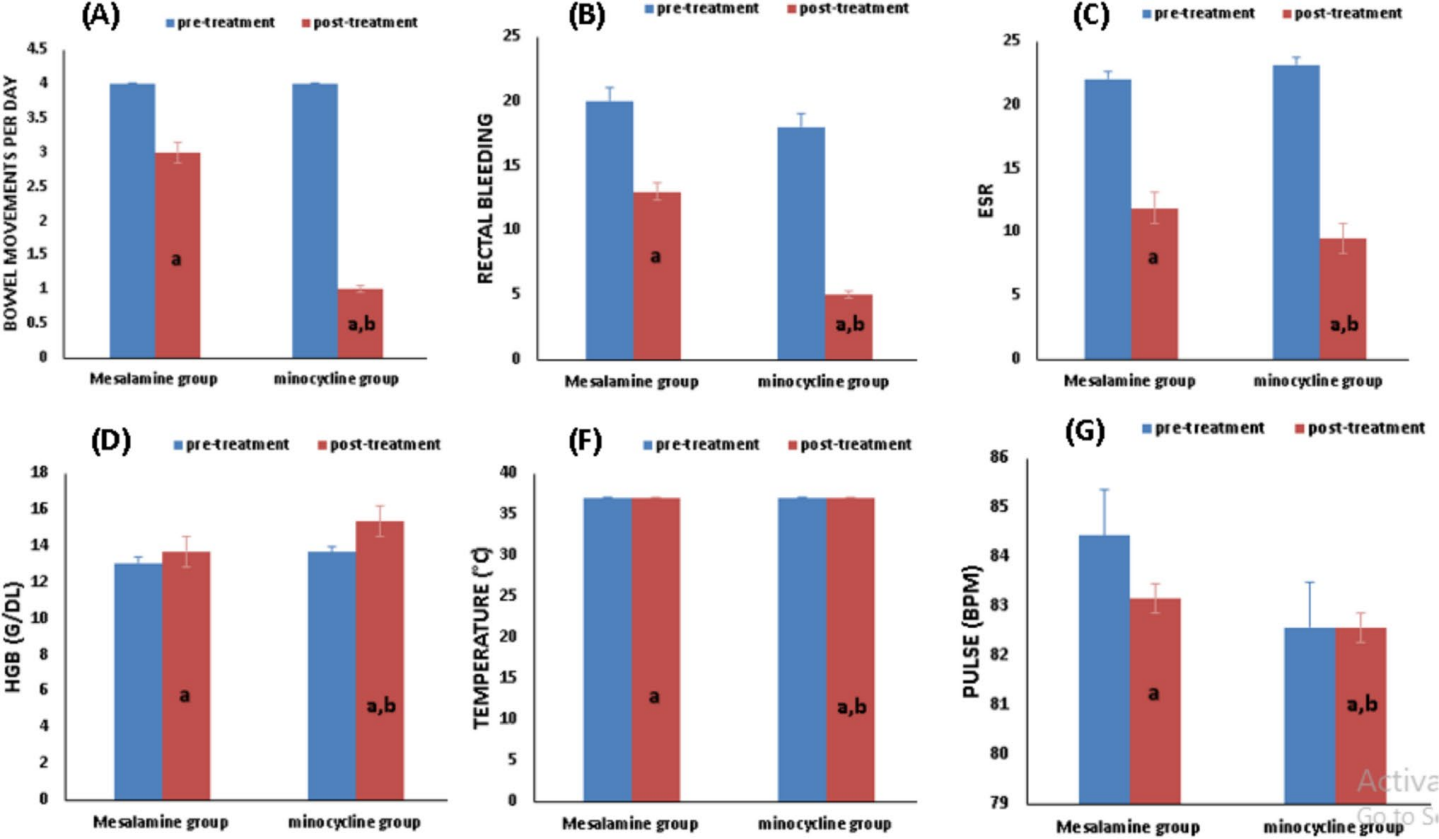
Effect of studied medication on Disease Severity according to the Modified Truelove and Witt*’*s Classification. Mesalamine group, ulcerative colitis patients treated with mesalamine; minocycline group, ulcerative colitis patients treated with mesalamine plus Minocycline. Effect of drug treatments on (**A**) changes in bowel movements per day, (**B**) rectal bleeding, (**C**) changes in ESR, (**D**) changes in Hgb, (**F**) changes in temperature, (**G**) changes in pulse before and after 6 months. ESR, erythrocyte sedimentation rate; Hgb, hemoglobin. (a) Significance compared to before treatment. (b) Significance compared to group on mesalamine group.

In comparison with baseline, paired *t*-test of the mesalamine and the minocycline group revealed a statistical decline in ESR (22.04 ± 4.194 versus 11.87 ± 3.238, *p* < 0.0001), as well as a substantial rise in Hgb (13.06 ± 1.031 versus 13.67 ± 0.9310, *p* = 0.0068). And no statistical significance in pulse (84.43 ± 4.660 versus 83.17 ± 5.123, *p* = 0.1091) (Table 3) and the minocycline group, statistical drop in ESR in comparison with baseline (23.13 ± 4.73versus 9.478 ± 2.55, *p* < 0.0001), in addition to a significant increase in Hgb (13.65 ± 1.327 versus 15.35 ± 1.297, *p* < 0.0001), and no statistical significance in pulse (83.17 ± 5.883 versus 82.57 ± 5.759, *p* = 0.1341) (Table 3 and Fig. 3).

**Table 3:** Effect of study medications on disease severity according to the modified Truelove and Witt’s classification

	Mesalamine group			Minocycline group			*p* value
Character	Before treatment	After treatment	*p* value	Before treatment	After treatment	*p* value	After treatment
**Bowel movements per day**	4 (2–5)	3 (1–3)	0.0027^a^	4 (3–5)	1 (1–2)	0.0001^a^	0.0198^b^
**Rectal bleeding**	20/23	13/23	0.0270^c^	18/23	5/23	0.0002	0.0157^d^
**ESR**	22.04 ± 4.194	11.87 ± 3.238	0.0001^a^	23.13 ± 4.73	9.478 ± 2.55	< 0.0001^a^	0.0080^b^
**Hgb (g/dl)**	13.06 ± 1.031	13.67 ± 0.9310	0.0068^a^	13.65 ± 1.327	15.35 ± 1.297	< 0.0001^a^	< 0.0001^b^
**Temperature (°C)**	37 (36.7–37.2)	37 (36.5–37.2)	0.1250^a^	37 (36.5–37)	37 (36.5–37)	0.2500^a^	0.0779^b^
**Pulse (bpm)**	84.43 ± 4.660	83.17 ± 5.123	0.1091^a^	83.17 ± 5.883	82.57 ± 5.759	0.1341^a^	0.7067^b^

Data was presented as number, mean ± SD, median, and interquartile range. Mesalamine group: UC patients treated with mesalamine. Minocycline group: UC patients treated with mesalamine plus minocycline

*ESR* erythrocyte sedimentation rate, *Hgb* hemoglobin

^a^Level of significance within the same group by paired *t*-test and Wilcoxon test for parametric and nonparametric data respectively

^b^Level of significance between groups using unpaired *t*-test and Mann–Whitney test for parametric and nonparametric data respectively

^c^Level of significance within a group using the McNamar test

^d^Level of significance between groups using the chi-square test

Significance at *p* < 0.05

After 6 months of intervention, an unpaired *t*-test evaluating both groups revealed significant change in ESR and Hgb and no significant difference in pulse, as follows: ESR (*p* = 0.0080), Hgb (*p* < 0.0001), and pulse (*p* = 0.7067) (Table 3 and Fig. 3).

Analysis using the Wilcoxon signed-rank test revealed that the median values of the assessed parameters in the mesalamine group were significantly lower after treatment compared to their initial baseline medians: bowel movements per day [4(2–5) versus 3(1–3) (*p* = 0.0027)], and no statistical significance in temperature [37 (36.7–37.2) versus 37 (36.5–37.2) (*p* = 0.1250)] (Table 3).

The Wilcoxon test showed a significant drop in the median of the following parameters when comparing the minocycline group to the baseline: bowel movements per day [4(3–5) versus 1(1–2) (*p* < 0.0001)], and no statistical significance in temperature [37 (36.5–37) versus 37 (36.5–37) (*p* = 0.2500)] (Table 3).

After 6 months of intervention, statistically significant changes were observed in bowel movements per day (*p* = 0.0198) and no statistical significance in temperature (*p* = 0.0779) as indicated by the Mann–Whitney test (Table 3).

Treatment with mesalamine resulted in a significant decrease in the proportion of patients with bleeding (McNemar test, *p* = 0.027; 20 vs. 13). A statistically more substantial reduction in bleeding was seen in the minocycline group (McNemar test, *p* = 0.0002; 18 vs. 5). Overall, the incidence of bleeding varied significantly between the two treatment groups (chi-square test, *p* = 0.0157) (Table 3 and Fig. 3).

### Minocycline and mesalamine’s effects on serum biomarkers

**Fig. 4:**
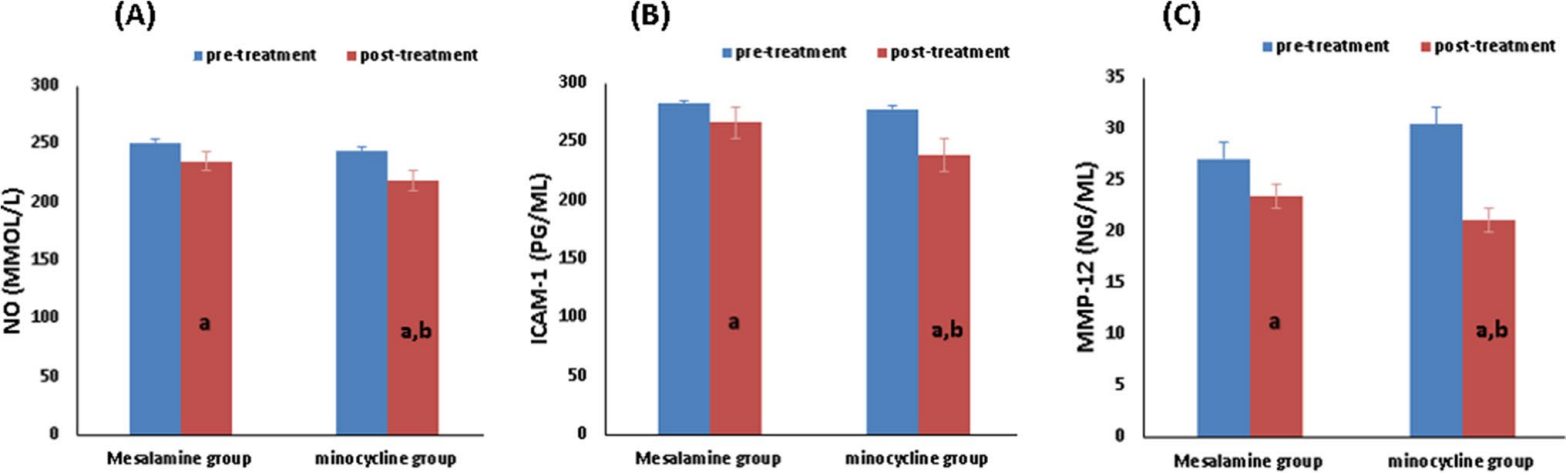
Effect of studied medication on serum parameters. Mesalamine group, ulcerative colitis patients treated with mesalamine; minocycline group, ulcerative colitis patients treated with mesalamine plus minocycline. Effect of drug treatments on (**A**) changes in serum NO level, (**B**) changes in serum ICAM-1 level, and (**C**) changes in serum MMP-12 level, before and after 6 months. NO Nitric oxide, ICAM-1 intracellular adhesion molecule-1, and MMP-12 Matrix metalloproteinases. (a) Significance compared to before treatment. (b) Significance compared to group on mesalamine group

Baseline values were similar between the groups (*p* > 0.05, unpaired *t*-test). However, within the mesalamine group, a paired *t*-test indicated a significant reduction in the measured parameters after treatment compared to their initial values, as follows: NO (250.6 ± 20.73 versus 235.1 ± 23.66, *p* = 0.0015), ICAM-1 (282.1 ± 10.69 versus 266.1 ± 21.78, *p* = 0.0025), and MMP-12(27.07 ± 3.822 versus 23.49 ± 3.718, *p* = 0.0005) (Table 4 and Fig. 4).

**Table 4:** Effect of study medications on serum parameters

	Mesalamine group			Minocycline group			*p* value
Character	Before treatment	After treatment	*p* value	Before treatment	After treatment	*p* value	After treatment
**NO (μmol/L)**	250.6 ± 20.73	235.1 ± 23.66	0.0015^a^	244 ± 22.69	218.3 ± 27.84	0.0087^a^	0.0323^b^
**ICAM-1 (pg/ml)**	282.1 ± 10.69	266.1 ± 21.78	0.0025^a^	278 ± 11.14	238.5 ± 18.4	0.0001^a^	0.0001^b^
**MMP-12 (ng/ml)**	27.07 ± 3.822	23.49 ± 3.718	0.0005^a^	30.44 ± 4.543	21.13 ± 4.313	0.0001^a^	0.0270^b^

Data were presented as mean ± SD; mesalamine group: ulcerative colitis patients treated with mesalamine; minocycline group: ulcerative colitis patients treated with mesalamine plus Minocycline

*NO* nitric oxide, *ICAM-1* intracellular adhesion molecule-1, *MMP-12* matrix metalloproteinases

^a^Level of significance within the same group by paired *t*-test

^b^Level of significance between groups using unpaired *t*-test

Significance at *p* < 0.05

The following parameters statistically declined in the minocycline group when compared to the baseline, according to a paired *t*-test: NO (244 ± 22.69 versus 218.3 ± 27.84, *p* = 0.0087), ICAM-1 (278 ± 11.14 versus 238.5 ± 18.4, *p* = 0.0001), and MMP-12 (30.44 ± 4.543 versus 21.13 ± 4.313, *p* = 0.0001) (Table 4).

All of the tested markers changed significantly after 6 months of intervention, according to the unpaired *t*-test between each of the groups, as follows: NO (*p* = 0.0323), ICAM-1 (*p* = 0.0001), and MMP-12 (*p* = 0.0270) (Table 4 and Fig. 4).

### Evaluation of drug-related side effects among the groups under study

During the course of this study, side effects associated with minocycline use were directly observed among participants. These included dental discoloration, dizziness, skin rash, and nausea. Although these adverse effects were generally mild, it is noteworthy that dental discoloration is typically reversible upon discontinuation of the drug. Initial side effects such as nausea, dizziness, or skin changes may occur, but many of these reactions tend to diminish over time as the body adapts to the medication. With appropriate monitoring and dosage adjustments, most side effects gradually subside during the course of treatment.

The two groups did not show any significant variation in the occurrence of these side effects, as shown in Table 5: dental discoloration (*p* = 0.233), dizziness (*p* = 0.6652), skin rash (*p* = 0.7381), and nausea (*p* = 0.7222).

**Table 5:** Analysis of drug-related side effects between the studied groups

Side effect	Mesalamine group	Minocycline group	*p* value
Dental discoloration	0 (0.00%)	3 (13.04%)	0.2333
Dizziness	2 (8.70%)	4 (17.39%)	0.6652
Skin rash	5 (17.39%)	7 (30.43%)	0.7381
Nausea	6 (26.09%)	4 (17.39%)	0.7222

Data was presented as numbers and percentages. Mesalamine group: ulcerative colitis patients treated with mesalamine; minocycline group: ulcerative colitis patients treated with mesalamine plus minocycline. Significance at *p* < 0.05 using Fisher or chi-square test as appropriate

### Analysis of correlations among the biomarkers under study

A significant inverse relationship between PMS and SIBD score (*r* =  − 0.7378, *p* < 0.0001) indicated that higher disease activity (higher PMS) was associated with lower quality of life (lower SIBD). Similarly, SIBD scores showed a significant inverse correlation with ICAM-1 levels (*r* =  − 0.6959, *p* < 0.0001) and with pain intensity as measured by the BPI (*r* =  − 0.8555, *p* < 0.0001), suggesting that better quality of life was associated with lower ICAM-1 and lower pain intensity. In contrast, a significant positive correlation was found between ICAM-1 and MMP-12 levels (*r* = 0.4703, *p* < 0.0001), indicating that higher levels of one were associated with higher levels of the other.

## Discussion

As a type of IBD, UC primarily manifests with gastrointestinal symptoms, including diarrhea, abdominal pain, bloody stools mixed with mucus, and tenesmus (urgency to defecate). UC is an incurable and recurrent condition that can ultimately progress to colon cancer (Zhang and Shi 2020). Despite numerous studies linking UC to multiple elements, including genetic predispositions, environmental influences, immune response, and infections, the exact cause remains unclear (Seyed Tabib et al. 2020; Le Berre et al. 2023). The main approach to managing UC is medical treatment, which seeks to achieve and maintain remission, enhance the patient’s quality of life, and decrease the need for colectomy (Gros and Kaplan 2023).

It is important to note that this is the first clinical trial investigating the impact of minocycline as an additional therapy for individuals with mild to moderate UC. Considering the absence of significant disparities in demographic or clinical characteristics between the groups at baseline, coupled with the absence of drug interaction between minocycline and mesalamine, as distinct enzymes metabolize them (Klotz and Maier 1987), implies that the positive treatment outcomes observed were primarily attributable to the administered medications.

This clinical study delves into the effects of minocycline in UC patients, offering important new insights into its therapeutic promise for this condition. We found that combining minocycline with mesalamine substantially reduced the PMS and pain intensity of BPI scores and significantly improved patients’ quality of life through improving domains of SIBDQ, and significantly decreased all criteria of Truelove and Witts DAI including bowel movement, rectal bleeding, hemoglobin, and ESR apart from no significant difference in temperature and pulse. This outcome can be directly attributed to the combined anti-inflammatory properties of minocycline and mesalamine. These findings aligned with previous reports that examined the impact of minocycline in animal colitis (Garrido-Mesa et al. 2011; Huang et al. 2009; Vezza et al. 2023). It is well known that UC negatively impacts HRQL and carries a significant economic cost. Our finding of significantly lower SIBDQ scores in UC patients is consistent with the research by Hoivik et al. (2012), which showed that UC patients experienced a decline in HRQL compared to the broader Norwegian population. A separate investigation demonstrated that minocycline elicited a significant reduction in inflammatory biomarkers and attenuated inflammation, histopathological alteration, and apoptosis in animal models of colitis (Garrido-Mesa et al. 2011; Vezza et al. 2023). Additionally, our results support prior research on adjunctive anti-inflammatory medications in IBDs, which found that pentoxifylline and metformin protect the colon and boost the effectiveness of mesalamine (Bahaa et al. 2024; El‑Haggar et al. 2024).

Patients in the mesalamine group of this study experienced significant improvements across several measures, including a reduction in disease activity (PMS and DAI), decreased pain intensity (BPI), lower levels of inflammatory markers (serum NO, ICAM-1, MMP-12), and a notable increase in their quality of life (SIBDQ) compared to pre-treatment levels. These improvements can be attributed to mesalamine itself. It is regarded as the first-line therapy for managing mildly to moderately active UC, for both inducing and maintaining remission (D’amico et al. 2024). Although its specific mode of action is unknown, it is assumed to function locally inside the intestinal lumen by inhibiting the formation of numerous inflammatory mediators such as prostaglandins and leukotrienes (Meier and Sturm 2011). Furthermore, it is likely to exert its effects, in part, by acting as a biological antioxidant through the neutralization of oxygen-free radicals (Ahnfelt-Rønne et al. 1990). This proposed mechanism is supported by a study that examined the influence of mesalamine on NO, ICAM-1, and MMP-12 in the context of experimental colitis (Kennedy et al. 1999; Ford et al. 2011).

Based on the recent findings, the minocycline group demonstrated notably reduced serum ICAM-1 levels in comparison to both the mesalamine group and the initial baseline levels. These outcomes align with several studies that found elevated ICAM-1 levels in the colon’s mucosal layer in experimental colitis models, which were reduced following minocycline treatment. This effect contributes to the migration of inflammatory cells into the colon’s tissue, exacerbating inflammation in UC (Garrido-Mesa et al. 2011; Vainer 2010). Inhibiting the interaction between TNFR1 (a receptor for tumor necrosis factor-α) and TRAF2 (TNF receptor–associated factor 2) (a protein that mediates intracellular signal transduction) represents a critical early step in the TNF-α signaling pathway. By blocking this interaction, minocycline prevents the subsequent activation of the nuclear factor kappa B (NF-κB) pathway. Under normal conditions, TNF-α triggers NF-κB activation through the phosphorylation of IκBα (inhibitor of NFκB), which results in the release and nuclear translocation of NF-κB-p65. Once in the nucleus, NF-κB initiates the transcription of various genes, including ICAM1, a key protein involved in inflammation and cell adhesion. So, minocycline disrupts this entire NF-κB activation cascade, ultimately resulting in decreased levels of ICAM1 and attenuation of the inflammatory response (Weiler and Dittmar 2019).

In the minocycline group, the level of NO was significantly reduced in this study, both relative to the mesalamine group and their starting levels. This finding aligns with previous researches (Garrido-Mesa et al. 2011; Amin et al. 1996). Elevated NO synthesis within the colon, particularly when facilitated by iNOS, is involved in the development of IBD and UC (Farzaei et al. 2024). Several experimental studies have shown that minocycline affects NO (Mousavi et al. 2016; Huang et al. 2009). Nonetheless, this study is the first to provide clinical evidence that minocycline effectively reduces serum NO in patients with UC. The mechanism by which minocycline reduces serum NO is likely through indirect pathways, such as the suppression of iNOS expression, which in turn decreases serum NO levels. iNOS is crucial in regulating the release of pro-inflammatory cytokines. Its activation can trigger the mitogen-activated protein kinase (MAPK) cascade (Lin et al. 2001), which boosts the function of transcription factors such as activator protein-1 (AP-1) and NF-κB. NF-κB activation is linked to elevated production of pro-inflammatory cytokines, including interleukin-6 (IL-6) and tumor necrosis factor-alpha (TNF-α). The AP-1 family works alongside NF-κB to further stimulate TNF-α transcription. Previous studies indicate that NO may also amplify the activation of AP-1 triggered by cytokine activation through cyclic-GMP-dependent protein kinase, thereby implying that NO can directly and indirectly affect pro-inflammatory cytokine gene activation. Additionally, NO and oxidative stress can directly influence TNF-α production by activating the TNF-α-converting enzyme, which increases TNF-α release (Kankuri et al. 2003). Furthermore, minocycline could directly block NO by inhibiting NO-induced p38 MAP kinase phosphorylation (Lin et al. 2001). Huang et al. showed that by inhibition of NO release, minocycline reduces inflammation in mice associated with DSS- and TNBS-induced UC (Huang et al. 2009).

In the present study, the levels of MMP-12 in the minocycline group were found to be significantly lower compared to both the mesalamine group and the initial baseline measurements. These promising results aligned with previous studies (Nighot et al. 2021; Suzuki et al. 2010). Nighot et al. (2021) reported the role of MMP-12 and macrophages in mouse colonic tight junction (TJ) permeability and DSS-induced colitis. Matrix metalloproteinases (MMPs) are enzymes that degrade ECM. They are categorized by their target substrates into groups like gelatinases [MMP-2, −9], collagenases [MMP-1, −8, −13, −18], elastase [MMP-12], stromelysins [MMP-3, −7, −10, −11, −19], and membrane type [MMP-1, −5] (Ravi et al. 2007). MMP-12, an inflammatory mediator, is essential for macrophage movement across the intestinal epithelium, increasing TJ permeability. Macrophages are vital for the upkeep of intestinal equilibrium and the control of inflammation. In a healthy state, they eliminate apoptotic cells and harmful microorganisms without causing inflammation, producing immunoregulatory cytokines. During inflammation, inflammatory macrophages accumulate and release high levels of pro-inflammatory cytokines TNF-α and IL-1β, contributing to intestinal inflammation seen in UC. Furthermore, macrophage transmigration mediated by MMP-12 increases the permeability of tight junctions and is linked to the degradation of basement membrane laminin. Minocycline’s anti-inflammatory action involves suppressing the accumulation of neutrophils and macrophages in the colon, as well as reducing the intestinal synthesis of key pro-inflammatory mediators IL-1β, TNF-α, and IL-6 (Huang et al. 2009). Also, its protective effects on the blood–brain barrier might be attributed to its potential to suppress MMP activity and enhance the levels of tight junction proteins (TJPs) (Zhao et al. 2023). Hu et al. have shown that synthetic agents, which reduce MMP mRNA expression and/or enzymatic function, can be effective therapies for diverse inflammatory diseases studied experimentally and in clinical practice. Some MMP inhibitors have already been used in clinical settings to manage inflammatory conditions. For instance, collagenase inhibitors like doxycycline hyclate and AZD8955 have been used to treat gum problems and osteoarthritis, respectively. Apratastat has been used to treat rheumatoid arthritis by restraining MMP-1, MMP-9, and MMP-13, and an MMP-12 blockage has been explored as a therapeutic approach for multiple sclerosis (Hu et al. 2007). Additionally, lower MMP-12 levels coincided with better histological outcomes in Crohn’s disease patients (Di Sabatino et al. 2009), indicating that minocycline might contribute to UC protection by downregulation of MMP-12.

Our study has some strength points, such as being the first randomized clinical study to investigate the adjunctive role of minocycline as add-on therapy to the standard treatment in patients with mild to moderate UC. Furthermore, we assessed the role of minocycline on UC through two approaches: clinically using PMS, BPI, and SIBDQ, and mechanistically by analyzing various mediators like NO, ICAM-1, and MMP-12.

Despite encouraging outcomes, this trial had some limitations that must be considered: First, a placebo control group was not included in the study design. As this was a pilot trial primarily aimed at assessing feasibility and preliminary efficacy, an active comparator design was used. It allowed us to assess the additive effect of minocycline over standard mesalamine therapy. Future randomized controlled trials should incorporate placebo groups for more rigorous evaluation. Second, endoscopic and histological assessments were not performed. Due to resource limitations, the study relied on clinical and biochemical markers to evaluate disease activity, which are non-invasive and suitable for early-phase investigations. However, mucosal healing is a critical endpoint in UC, and future studies should include endoscopic and histological evaluations to provide a more comprehensive assessment. Third, the study was conducted over a 6-month period, which limits our ability to assess the long-term efficacy and safety of minocycline in maintaining remission or preventing disease relapse, and no extended follow-up was planned, as this was a pilot trial to assess the possibility and initial efficacy. Future studies should include longer-term monitoring to evaluate the durability of the treatment effect and identify any delayed or cumulative safety concerns. Fourth, the study population was a small sample size limited to Egyptian patients, which may restrict the external validity and generalizability of the findings to broader, more diverse populations. To confirm these preliminary results and enhance their applicability across different demographic and geographic groups, larger multicenter studies involving varied populations are recommended. Finally, although microbiota analysis was not included in the current study, previous research indicates that minocycline may influence gut microbial composition, particularly under inflammatory conditions. This potential modulation of the microbiota could contribute to its observed anti-inflammatory and immunomodulatory effects in DSS-induced colitis in mice (Vezza et al. 2023). While our study primarily focused on clinical and biochemical outcomes, future research should incorporate microbiome profiling to better elucidate the role of microbial alterations in treatment response and disease progression.

## Conclusion

The findings from this randomized controlled pilot study indicate that combining minocycline with mesalamine is more effective than using mesalamine alone for treating mild to moderate UC. This enhanced efficacy may be attributed to the synergistic anti-inflammatory effects of the combination, which also reduces serum levels of NO, ICAM-1, and MMP-12. Additionally, it significantly improves the quality of life.

## Data Availability

Data will be made available on request.
